# Genome dynamics of high-risk resistant and hypervirulent *Klebsiella pneumoniae* clones in Dhaka, Bangladesh

**DOI:** 10.3389/fmicb.2023.1184196

**Published:** 2023-05-25

**Authors:** Arif Hussain, Razib Mazumder, Abdullah Ahmed, Umme Saima, Jody E. Phelan, Susana Campino, Dilruba Ahmed, Md Asadulghani, Taane G. Clark, Dinesh Mondal

**Affiliations:** ^1^Laboratory Sciences and Services Division, International Centre for Diarrhoeal Disease Research, Bangladesh (icddr,b), Dhaka, Bangladesh; ^2^Department of Infection Biology, London School of Hygiene and Tropical Medicine, London, United Kingdom; ^3^Clinical Microbiology and Immunology Laboratory, Laboratory Sciences and Services Division, International Centre for Diarrhoeal Disease Research, Bangladesh, Dhaka, Bangladesh; ^4^Biosafety and BSL3 Laboratory, Biosafety Office, International Centre for Diarrhoeal Disease Research, Bangladesh, Dhaka, Bangladesh

**Keywords:** Hypervirulent *Klebsiella pneumoniae*, high risk *Klebsiella pneumoniae* clone, *Klebsiella pneumoniae* from low and middle income country, omics, molecular epidemiology

## Abstract

*Klebsiella pneumoniae* is recognized as an urgent public health threat because of the emergence of difficult-to-treat (DTR) strains and hypervirulent clones, resulting in infections with high morbidity and mortality rates. Despite its prominence, little is known about the genomic epidemiology of *K. pneumoniae* in resource-limited settings like Bangladesh. We sequenced genomes of 32 *K. pneumoniae* strains isolated from patient samples at the International Center for Diarrhoeal Disease Research, Bangladesh (icddr,b). Genome sequences were examined for their diversity, population structure, resistome, virulome, MLST, O and K antigens and plasmids. Our results revealed the presence of two *K. pneumoniae* phylogroups, namely KpI (*K. pneumoniae*) (97%) and KpII (*K. quasipneumoniae*) (3%). The genomic characterization revealed that 25% (8/32) of isolates were associated with high-risk multidrug-resistant clones, including ST11, ST14, ST15, ST307, ST231 and ST147. The virulome analysis confirmed the presence of six (19%) hypervirulent *K. pneumoniae* (hvKp) and 26 (81%) classical *K. pneumoniae* (cKp) strains. The most common ESBL gene identified was *bla*_CTX-M-15_ (50%). Around 9% (3/32) isolates exhibited a difficult-to-treat phenotype, harboring carbapenem resistance genes (2 strains harbored *bla*_NDM-5_ plus *bla*_OXA-232_, one isolate *bla*_OXA-181_). The most prevalent O antigen was O1 (56%). The capsular polysaccharides K2, K20, K16 and K62 were enriched in the *K. pneumoniae* population. This study suggests the circulation of the major international high-risk multidrug-resistant and hypervirulent (hvKp) *K. pneumoniae* clones in Dhaka, Bangladesh. These findings warrant immediate appropriate interventions, which would otherwise lead to a high burden of untreatable life-threatening infections locally.

## Highlights

-*Klebsiella pneumoniae* is a major contributor of antimicrobial resistance crisis worldwide.
-It is a major public health threat presenting a therapeutic challenge.
-Despite its prominence.
-There is an acute scarcity of data on the major lineages within *K. pneumoniae* isolates from Bangladesh which has high infectious disease burden.
-We report the presence of hypervirulent Klebsiella pneumoniae (hvKp) with high virulence score, in addition to classical K. pneumoniae (cKp) isolates.
-We have also reported the predominance of international high-risk multidrug-resistant clones that were associated with high AMR rates.
-In addition to these strains, we identified strains with difficult-to-treat (DTR) resistance phenotypes that were completely resistant to all first line antimicrobial agents.
-Results of this study has set the ground for further whole genome-based surveillance studies to closely monitor the evolutionary trends in *K. pneumoniae* in Bangladesh that would help in initiating stringent actions for the development of control strategies.

## Introduction

*Klebsiella pneumoniae* is a notorious pathogen that has been implicated in a high number of high-risk infections and higher rates of resistance to multiple antimicrobial agents, including the last resort drugs (Paczosa and Mecsas, 2016). *K. pneumoniae* is a significant human pathogen acknowledged by the World Health Organization (WHO) and the US Centers for Disease Control and Prevention (CDC) as one of the priority pathogens exhibiting high propensities to acquire and spread resistance to multiple antimicrobial classes (Holt et al., 2015; Wyres et al., 2020). It is responsible for a wide range of hospital-acquired and community-acquired infections, including pneumonia, meningitis, wound infections, bacteremia, gastrointestinal and urinary tract infections (Musicha et al., 2019). The global spread of successful clonal lineages of *K. pneumoniae* is associated with the spread of extended-spectrum β-lactamase (ESBLs) and carbapenemases (Magiorakos et al., 2012). It is a major public health threat, presenting an extreme therapeutic challenge (Magiorakos et al., 2012). Classical *K. pneumoniae* (cKP) strains are primarily restricted in causing infections among immunocompromised patients (Paczosa and Mecsas, 2016). However, the emergence and rapid dissemination of hypervirulent (hvKP) strains have broadened the scope of the susceptible population, including healthy and immunocompetent individuals (Paczosa and Mecsas, 2016). Compared to the classical (cKP) strains, the hvKP strains are mostly associated with infections that originate from the community (Yu et al., 2006). The virulence in the hvKP is contributed by the capsule (K1, K2, K20 capsular types), lipopolysaccharide (LPS) (*rmpA* and *rmpA2* regulatory genes), and siderophores (aerobactin) (Russo and Marr, 2019). The hvKP strains have further evolved by acquiring carbapenem resistance; these are referred to as carbapenem-resistant hypervirulent *K. pneumoniae* -hvKP (CR-hvKp) (Okanda et al., 2021; Sundaresan et al., 2022; Hussain et al., 2023). They are responsible for a serious public health crisis as they do not just represent hypervirulence and multi-drug resistance but exhibit high rates of transmission (Sundaresan et al., 2022). Genomic analysis of such strains remains an active area of research. *K. pneumoniae* are usually classified by their sequence types (STs); strains of certain STs have expanded clonally and distributed worldwide. They are called high-risk clones (Wyres and Holt, 2016; Wyres et al., 2019). Bacterial whole-genome sequencing studies have identified distinct phylogroups associated with *K. pneumoniae*; they include KpI, KpII and KpIII. These have been later classified as individual species- KpI as *K. pneumoniae*, KpII as *K. quasipneumoniae* and KpIII as *K. variicola* (Holt et al., 2015). The conventional microbiological methods are unable to distinguish between these *Klebsiella* species (Saxenborn et al., 2021).

Recent WGS studies on global collection of strains from hospital outbreaks and community-acquired *K. pneumoniae* infections provided a genomic framework for clonal diversity, antimicrobial resistance and virulence factors associated with this pathogen (Bowers et al., 2015; Holt et al., 2015; Fostervold et al., 2022). These studies have identified a wide spectrum of diversity, reinforced the diversification of *K. pneumoniae* into three phylogroups; KpI, KpII and KpIII and identified the role of hypervirulent and MDR clonal groups such as CG258, CG307, CG14 and CG15 in the nationwide and global dissemination of ESBL and carbapenem resistance (Bowers et al., 2015; Chung The et al., 2015; Holt et al., 2015; Fostervold et al., 2022). A nationwide AMR surveillance study in Bangladesh reported *K. pneumoniae* to be the third most abundant organism recovered from the clinical specimens, preceded by *E. coli* and *pseudomonas* species (Habib et al., 2021). Other in-country reports suggest the proportion of MDR *K. pneumoniae* has increased to over 80% (Ahmed et al., 2019; Aminul et al., 2021; Habib et al., 2021); this has driven the use of carbapenem as a drug of choice for treating MDR *K. pneumoniae* infections (Habib et al., 2021). Consequently, high carbapenem resistance rates were also reported from Bangladesh (Habib et al., 2021). In one study, up to 90% of isolates were found resistant to imipenem (Habib et al., 2021). Another study on a collection of isolates from a tertiary hospital in Dhaka, Bangladesh, reported the emergence of colistin resistance in a successful *K pneumoniae* sequence type 15 (Farzana et al., 2020). A study conducted by icddr,b between 2014 and 2017 showed that the increase in *K. pneumoniae* resistance rates has directly impacted the mortality in young children suffering concomitantly from bacteremia and pneumonia (Chisti et al., 2021).

Despite the increasing number of studies on *K. pneumoniae* nationally and globally, there is limited know-how on the genomic characteristics of such isolates from Bangladesh, as few studies have included the isolates from this region. Most in-country studies have analyzed hospital outbreak isolates focusing on MDR phenotypes and characterized them using conventional methods. Thus, there is a need for high-resolution studies on *K. pneumonia* isolates, including both resistant and susceptible ones. This would improve our understanding of population dynamics of community origin *K. pneumonia* beyond a handful of well-known clones.

This study used clinical *K. pneumoniae* isolates cultured from urine and pus specimens from a referral diagnostic center (icddr,b) in Dhaka, Bangladesh. We used a genomics-based approach to analyze the genetic diversity/relatedness, antimicrobial resistance and the virulome of the 32 clinical *K. pneumoniae* isolates to elucidate the genome dynamics of *K. pneumoniae* isolates, identifying the dominant/significant clones and evaluating their association with clinically relevant AMR and virulence determinants.

## Materials and methods

### Clinical isolates

We employed 32 *K. pneumoniae* isolates in this study. These were collected from a referral clinical microbiology laboratory at the International Center for Diarrheal Disease Research, Bangladesh (icddr,b), Dhaka, Bangladesh. The 32 *K. pneumoniae* isolates comprise 13 isolates recovered in August 2019, 12 isolates from September 2019 and 6 isolates from October 2019. The isolates from each month represent around 5% of *K. pneumoniae* isolates cultured from patient samples. The remaining one isolate archived at the Genome Center originating from the same setting was also used in this study. The isolates were randomly collected with minimum criteria that included: (i) Originating from different patients; (ii) Excluding samples from hospitalized patients; (iii) Phenotypically confirmed as *K. pneumoniae* by the Vitek-2 bacterial identification system. We employed disk diffusion tests to evaluate the antimicrobial susceptibility of the study isolates as per the CLSI guideline. The following 20 antibiotics were tested against the isolates: ampicillin, nitrofurantoin, ceftazidime, ceftriaxone, cefuroxime, cefixime, amikacin, ciprofloxacin, cotrimoxazole, nalidixic, cefepime, piperacillin/tazobactam, amoxycillin/clavulanic acid, gentamicin, tigecycline, cefoperazone/sulbactam, ertapenem, meropenem, imipenem and colistin. MDR was defined as resistance to at least one antimicrobial agent representing three antimicrobial classes. Difficult-to-treat (DTR) phenotype denotes resistance to all β-lactam generations, including carbapenems and fluoroquinolone. Extensively drug resistance (XDR) was defined as resistance to all antibiotic categories except to one or two antibiotic categories.

### Whole genome sequencing

Bacterial genomic DNA extraction was done from single-colony cultures using the QIAamp DNA MINI kit per the manufacturer’s instructions. DNA QC and quantification were determined using the NanoDrop spectrophotometer (Thermo Fisher Scientific, United States) and Qubit 2.0 Fluorometer (Life Technologies), respectively (Mazumder et al., 2020b). The library preparation was performed utilizing the Nextera XT DNA library preparation kit (Illumina). The normalized and pooled library was subjected to 150-base paired-end reads sequencing using the Mid-output v2.5 sequencing kit. Sequencing was performed at the icddr,b Genome Center using the Nextseq500 platform (Mazumder et al., 2021).

### Bioinformatic analysis

The high-quality reads were used for *de novo* assembly using SPAdes 3.11.1 (Bankevich et al., 2012). We performed the quality checks of the genome assemblies using QUAST 5.0.2 (Gurevich et al., 2013). The resulting genome assemblies were subjected to the Prokka 1.14.6 (Seemann, 2014) software for annotation.

The resistome of *K. pneumoniae* isolates was obtained by analyzing the presence of antimicrobial resistance genes and plasmid replicons using ResFinder 4.1 (Zankari et al., 2017) and plasmidFinder (Carattoli et al., 2014) hosted by the Center for Genomic Epidemiology (CGE). We used an identity cut-off of 98% and a coverage threshold of 80%.

Virulence genes were identified using a custom database of 28 *K. pneumoniae* virulence genes derived from the Virulence Factor Database (VFDB) (Chen et al., 2005) and known virulence genes from NCBI. Gene presence/absence was evaluated by BLASTn analysis with a cut-off coverage of 80% and a cut-off identity of 96%. Genes were classified functionally as; type I fimbriae (*fimD*, *fimK*, *fimH*, and *fimC*), *type* III fimbriae (mrkD, mrkJ, mrkF, mrkC, mrkA, and mrkI), siderophores/yersiniabactin (*iutA*, *entB*, *ybtS*, *iucA*, *ybtA*, *irp1*, *irp2*, and *fyuA*), regulator of mucoid phenotype [*p*-*rmpA* (accession No. KJ469368.1), *p*-*rmpA2* (accession No. S64176.1)], salmochelin (iroB, iroC, iroD, and iroN), allantoin utilization (*alls*), type IV pili (*pilW*), type IV secretion system (*clpV/tssH*), metabolite transporter [*peg-344* (accession No. MZ245622.1)] and colibactin [*pks* gene cluster (accession No. AM229678.1)]. Isolates were defined as hypervirulent (hvKp) *K. pneumoniae* if positive for ≥1 biomarkers including; *peg-344*, *iroB*, *iucA*, *rmpA*, and *rmpA2* as suggested by the report from hypervirulent *K. pneumoniae* investigator group (Russo et al., 2018).

Additionally, isolates were typed using the Kaptive database (Wick et al., 2018) to identify the polysaccharide capsule (k antigen) and lipopolysaccharide (O antigen). The ST of each strain was assessed by submitting the genome assemblies to MLST 2.0 at Center for Genomic Epidemiology (CGE).

### Core and pan-genome analysis

We performed the core and pan-genome analysis of 32 *K. pneumoniae* genomes. The GFF files obtained from Prokka (Seemann, 2014) were used as input for Roary (Page et al., 2015). The default settings were employed: core-gene threshold, 99%; the maximum number of clusters, 50,000 and inflation value for the MCL algorithm was: 1.5. A multi-fasta alignment was created using PRANK (option-e in roary). RaxML (Stamatakis, 2014) was used for constructing a phylogenetic tree from the aligned fasta file generated by Roary. The GAMA distribution of rates were used to evaluate the final tree, the robustness of the clades was verified using 100 bootstrap repetitions. The core-genome phylogeny and pangenome fingerprints of the isolates, along with their metadata, were visualized by Phandango (Hadfield et al., 2018). Snippy (Bush et al., 2020) was used to find SNPs among the genomes. *Klebsiella pneumoniae* strain KP64 (NZ_AP018750.1) was used as a reference genome. Gubbins (Croucher et al., 2015) was used to filter true point mutations from those arising from recombination.

### Statistical analysis

Statistical comparisons between proportions were computed using Fisher’s exact two-tailed test. The non-parametric Mann–Whitney *U* test was used to calculate the statistical analysis for the aggregate virulence score. SPSS version 10.0 was used for all statistical calculations. Statistical significance was defined as *p* values ≤0.05.

## Results

### Population structure of *Klebsiella pneumoniae*

**Figure 1:**
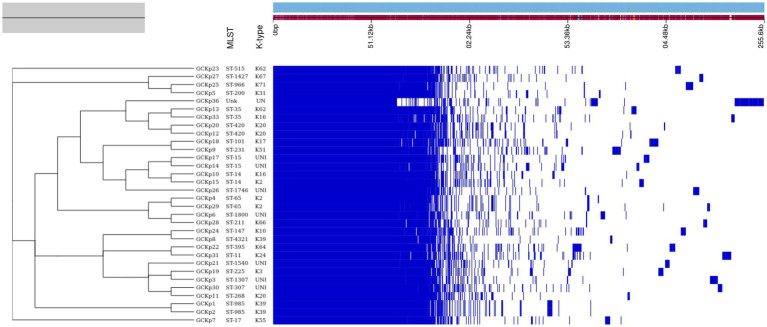
The core genome-based phylogenetic tree of 32 *K. pneumoniae* isolates compared to the metadata (STs and capsular polysaccharide) and a matrix with the presence and absence of core and accessory genes.

**Figure 2:**
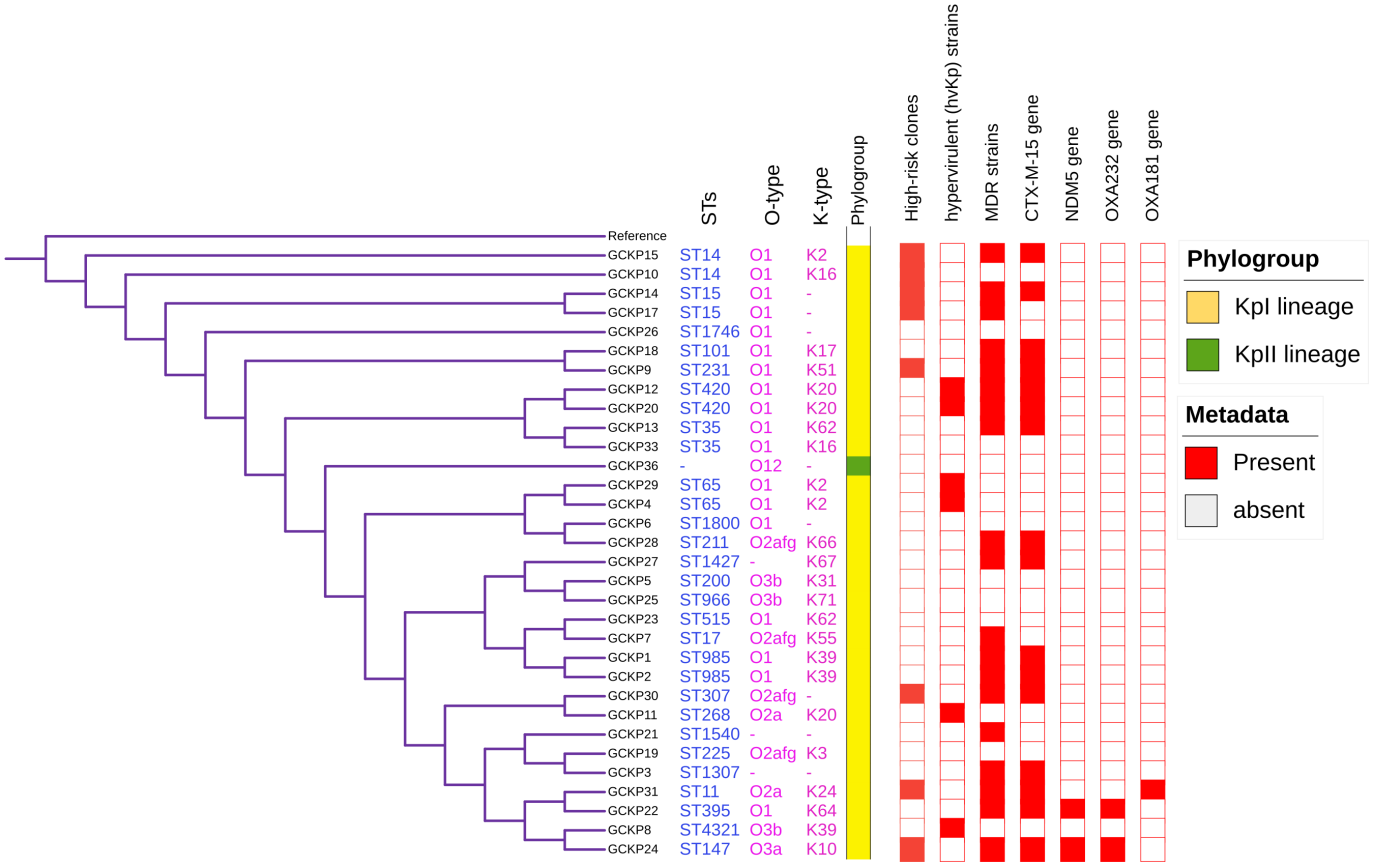
Core-genome SNP-based phylogenetic tree of 32 *K. pneumoniae* isolates analyzed from Dhaka, Bangladesh. The tree is annotated with the STs, O antigen, capsular polysaccharide, phylogroups, high-risk groups, hvKp strains, MDR status, presence of *bla*_CTX-M-15_, *bla*_NDM5_, *bla*_OXA232_, and *bla*_OXA181_ genes.

The genome features, associated metadata, and accession details are provided in Table 1. Pan-genome analysis of the 32 *K. pneumoniae* sequences predicted an average of 5,043 genes per genome, resulting in a pan-genome consisting of 12,778 genes. We identified 3,226 core and 9,552 accessory genes in our genome collection. A roary matrix was constructed from 32 *K. pneumoniae* genomes to demonstrate the genetic relatedness at the core genome level (Figure 1). A considerable correlation was observed between the phylogenetic clustering and some of the sequence types such as ST985, ST65, ST14, ST420, ST35 and ST15 and their K-types (K2, K20, K39 and K62) indicating presence of monophyletic clades corresponding to the above described STs. The strain GCKP36 exhibited the highest variation in core and accessory gene content compared to the rest of the genomes. WGS species identification confirmed that the strain GCKP36 (3%) was *K. quasipneumoniae* while the rest were *K. pneumoniae* (97%). The SNP analysis of 32 strains revealed 187,851 SNPs. The majority of Bangladeshi strains belonged to KpI phylogroup (*K. pneumoniae*) [97% (31/32)], whereas only one isolate was KpII (*K. quasipneumoniae*) [3% (1/32)]. None of the strains in our collection belonged to KpIII phylogroup (*Klebsiella variicola*). The isolate of KpII (GCKP36) exhibited the highest number of SNPs (221,163) compared to the rest of the strains belonging to KpI lineage. It showed the longest branch length (indicating its divergence from KpI strains); however, it did not form a separate branch with the rest of the genomes as the number of genomes belonging to this lineage was only one. Like the core-genome-based phylogeny, the core genome-SNP-based phylogenetic tree clustered the strains having identical STs associated with identical metadata, particularly STs such as ST420 and ST985 strongly correlated with the serogroups and MDR phenotype and strains belonging to ST420 and ST65 showed correlation with hypervirulence genotype. Strains belonging to ST35 did not show any substantial correlation (Figure 2). Surprisingly, the strains belonging to ST14 formed singletons. They were inconsistent with the serogroups and MDR phenotype data (Figure 2).

**Table 1:** Genome features and metadata of 32 *K. pneumoniae* isolates from Bangladesh.

Sl No.	Strain name	Accession number	Source	Isolation date	Genome coverage	Contig no. (>200 bp)	No. of Coding Sequences (CDS)	Genome Size
1	GCKp1	JACSGO000000000.1	Urine	17-Aug-2019	128.4X	53	5,224	5,464,874
2	GCKp2	JACSGN000000000.1	Urine	17-Aug-2019	129.1X	54	5,223	5,463,930
3	GCKp3	JACSGM000000000.1	Urine	19-Aug-2019	128.5X	72	5,230	5,433,644
4	GCKp4	JACSGL000000000.1	Urine	21-Aug-2019	150.8X	71	5,113	5,378,293
5	GCKp5	JACSGK000000000.1	Urine	19-Aug-2019	114.8X	46	4,906	5,157,139
6	GCKp6	JACSGJ000000000.1	Urine	19-Aug-2019	140.2X	97	5,180	5,378,586
7	GCKp7	JACSGI000000000.1	Urine	26-Aug-2019	97.3X	75	5,151	5,360,805
8	GCKp8	JACSGH000000000.1	Urine	25-Aug-2019	61.4X	42	4,910	5,173,664
9	GCKp9	JACSGG000000000.1	Urine	25-Aug-2019	160.4X	97	5,294	5,466,648
10	GCKp10	JACSGF000000000.1	Urine	25-Aug-2019	147.9X	57	5,111	5,362,783
11	GCKp11	JACSGE000000000.1	Urine	25-Aug-2019	148.9X	78	5,190	5,462,278
12	GCKp12	JACSGD000000000.1	Urine	30-Aug-2019	120.7X	91	5,287	5,513,067
13	GCKp13	JACSGC000000000.1	Urine	28-Aug-2019	108.3X	92	5,333	5,531,380
14	GCKp14	JACSGB000000000.1	Urine	1-Sep-2019	140X	96	5,203	5,477,494
15	GCKp15	JACSGA000000000.1	Urine	16-Sep-2019	130.9X	64	5,323	5,526,736
16	GCKp17	JACSFZ000000000.1	Urine	20-Sep-2019	124.2X	104	5,280	5,503,979
17	GCKp18	JACSFY000000000.1	Urine	18-Sep-2019	101.3X	80	5,354	5,509,272
18	GCKp19	JACSFX000000000.1	Urine	18-Sep-2019	143.4X	84	5,259	5,473,267
19	GCKp20	JACSFW000000000.1	Urine	25-Sep-2019	134.3X	89	5,378	5,593,466
20	GCKp21	JACSFV000000000.1	Urine	25-Sep-2019	118.9X	83	5,221	5,404,000
21	GCKp22	JACSFU000000000.1	Urine	25-Sep-2019	139.1X	109	5,574	5,682,493
22	GCKp23	JACSFT000000000.1	Urine	30-Sep-2019	123.5X	83	5,147	5,346,379
23	GCKp24	JACSFS000000000.1	Urine	30-Sep-2019	81.7X	91	5,365	5,513,547
24	GCKp25	JACSFR000000000.1	Urine	29-Sep-2019	151.8X	61	5,045	5,270,340
25	GCKp26	JACSFQ000000000.1	Urine	29-Sep-2019	159.4X	53	5,102	5,341,693
26	GCKp27	JACSFP000000000.1	Urine	4-Oct-2019	162.9X	74	5,184	5,377,405
27	GCKp28	JACSFO000000000.1	Urine	2-Oct-2019	168.1X	69	5,015	5,245,228
28	GCKp29	JACSFN000000000.1	Urine	7-Oct-2019	130.6X	73	5,169	5,437,274
29	GCKp30	JACSFM000000000.1	Urine	7-Oct-2019	167.7X	77	5,324	5,521,074
30	GCKp31	JACSFL000000000.1	Urine	13-Oct-2019	138.7X	121	5,536	5,655,436
31	GCKp33	JACSFK000000000.1	Urine	19-Oct-2019	132.5X	76	5,256	5,467,282
32	GCKp36	JACSFI000000000.1	Pus	15-Jun-2018	153.9X	103	5,163	5,362,244

### Isolates typing

The degree of *K. pneumoniae* sequence diversity was further analyzed by typing STs and surface-exposed polysaccharides (O and K antigens). We identified 26 diverse STs among 32 *K. pneumoniae* isolates in our collection that included six STs associated with high-risk clones, namely ST11 (3%), ST14 (6%), ST15 (6%), ST307 (3%), ST231 (3%) and ST147 (3%) (Figure 2). Overall, six STs represented more than one isolate which includes, ST985 (2/32), ST65 (2/32), ST14(2/32), ST420(2/32), ST35(2/32) and ST15 (2/32). The remaining 20 STs were represented by a single isolate, as shown in Figure 1. Overall, a total of six O antigens were detected; the most prevalent O type was O1 (56.2%), followed by O2afg (13%) and O2a (6.2%). Sixteen different K-serotypes were identified in all strains. K antigens that were represented in more than one isolate comprised K2 (9%), K20 (9%), K16 (6%) and K62 (6%) (Figure 2). Among these, the two capsular types, K2 and K20 were mainly associated with hypervirulence genotype. The remaining 12 capsular types were singletons (Figure 2).

### Antimicrobial resistance and resistome of *Klebsiella pneumoniae* strains

None of the 32 *K. pneumoniae* strains was completely susceptible to the 20 antibiotics tested. The *K. pneumoniae* isolates showed the highest resistance to ampicillin (100%), followed by nitrofurantoin (94%), ceftazidime (60%) and cefixime (59%). Moderately resistant to ceftriaxone (56%), cefuroxime (56%), ciprofloxacin (53%), cotrimoxazole (50%) and nalidixic acid (50%). A varying portion of isolates were resistant to cefepime (44%), piperacillin/tazobactam (37%), amoxycillin/clavulanic acid (31%), gentamicin (28%), tigecycline (25%), cefoperazone/sulbactam (25%) and amikacin (13%). The strains were least resistant to carbapenems [ertapenem, meropenem and imipenem (9% each)] and colistin (6%). Over half (56%) of the *K. pneumoniae* strains were multi-drug resistant (MDR).

Our WGS analysis identified a total of 65 unique acquired AMR genes conferring resistance to different classes of antimicrobial agents with an average of 11 genes per genome (Table 2).

**Table 2:** Distribution of AMR genes among 32 clinical *K. pneumoniae* isolates from Dhaka, Bangladesh.

AMR gene	Gene description	Resistance	Prevalence	
			*n*	%
*OqxA*	efflux pump	fluoroquinolones	32	100
*OqxB*	efflux pump	fluoroquinolones	32	100
*fosA*	metalloglutathione transferase	fosfomycin	30	93.8
*bla*_CTX-M-15_	ESBL	aminopenicillins, cephalosporins	16	50
*bla*_TEM-1B_	ESBL	aminopenicillins, cephalosporins	16	50
*dfrA14*	dihydrofolate reductase	trimethoprim	16	50
*aph(3″)-Ib*	aminoglycoside 3′-phosphotransferase	aminoglycoside	12	37.5
*Sul2*	sulphonamide resistance dihydropteroate synthase	sulphonamides/co-trimoxazole	12	37.5
*aph(6)-Id*	aminoglycoside O-phosphotransferase	aminoglycoside	11	34.4
*Sul1*	sulphonamide resistance dihydropteroate synthase	sulphonamides/co-trimoxazole	11	34.4
*aac(6′)-Ib-cr*	acetyltransferase	aminoglycoside, fluoroquinolones	10	31.3
*tet(A)*	tetracycline efflux	tetracycline efflux	8	25
*mph(A)*	repressor protein MphR(A)	macrolide	8	25
*bla*_OXA-1_	ESBL	aminopenicillins, cephalosporins	8	25
*bla*_SHV-28_	b-lactamase	aminopenicillins	8	25
*catB3*	chloramphenicol acetyltransferase	phenicol	8	25
*bla*_SHV-106_	ESBL	aminopenicillins, cephalosporins	7	21.9
*qnrB1*	plasmid-mediated quinolone resistance	fluoroquinolones	6	18.8
*aadA2*	tetracycline efflux	aminoglycoside	6	18.8
*aac(3)-IIa*	aminoglycoside N(3)-acetyltransferase	aminoglycoside	5	15.6
*Arr-3*	rifampicin ADP-ribosyltransferase (Arr)	rifampicin	4	12.5
*qnrS1*	plasmid-mediated quinolone resistance	fluoroquinolones	4	12.5
*erm(B)*	ribosomal methylase	macrolide	3	9.4
*bla*_SHV-11_	b-lactamase	aminopenicillins	3	9.4
*bla*_SHV-89_	b-lactamase	aminopenicillins	3	9.4
*bla_SHV-56_*	ESBL	aminopenicillins, cephalosporins	3	9.4
*bla*_SHV-85_	b-lactamase	aminopenicillins	3	9.4
*bla*_SHV-79_	b-lactamase	aminopenicillins	3	9.4
*rmtF*	16S rRNA methyltransferase	aminoglycoside	3	9.4
*bla*_SHV-187_	ESBL	aminopenicillins, cephalosporins	2	6.25
*bla*_SHV-110_	ESBL	aminopenicillins, cephalosporins	2	6.3
*bla*_SHV-81_	beta-lactamase	aminopenicillins	2	6.3
*qnrS13*	plasmid-mediated quinolone resistance	fluoroquinolones	2	6.3
*bla*_SHV-67_	b-lactamase	aminopenicillins	2	6.3
*bla*_SHV-40_	ESBL	aminopenicillins, cephalosporins	2	6.3
*bla*_SHV-172_	b-lactamase	aminopenicillins	2	6.3
*aph(3′)-Ia*	aminoglycoside 3′-phosphotransferases	aminoglycoside	2	6.3
*bla*_SHV-75_	b-lactamase	aminopenicillins	2	6.3
*catA1*	chloramphenicol acetyltransferase	Phenicol	2	6.3
*bla*_NDM-5_	carbapenemase	cephalosporins, carbapenems	2	6.3
*bla*_OXA-232_	carbapenemase	cephalosporins, carbapenems	2	6.3
*bla*_SHV-182_	ESBL	aminopenicillins, cephalosporins	2	6.3
*armA*	aminoglycoside resistance methylase	aminoglycoside	2	6.3
*dfrA5*	dihydrofolate reductase	trimethoprim	1	3.1
*bla*_SHV-185_	b-lactamase	aminopenicillins	1	3.1
*bla*_SHV-94_	b-lactamase	aminopenicillins	1	3.1
*bla*_SHV-96_	b-lactamase	aminopenicillins	1	3.1
*dfrA15*	dihydrofolate reductase	trimethoprim	1	3.1
*floR*	florfenicol resistance gene	phenicol	1	3.1
*bla*_SHV-13_	b-lactamase	aminopenicillins	1	3.1
*bla*_SHV-70_	b-lactamase	aminopenicillins	1	3.1
*fosA5*	metalloglutathione transferase	fosfomycin	1	3.1
*bla*_SHV-33_	b-lactamase	aminopenicillins	1	3.1
*dfrA27*	dihydrofolate reductase	dihydrofolate reductase	1	3.1
*bla*_SHV-27_	ESBL	aminopenicillins, cephalosporins	1	3.1
*bla*_SHV-145_	ESBL	aminopenicillins, cephalosporins	1	3.1
*bla*_SHV-179_	b-lactamase	aminopenicillins	1	3.1
*bla*_SHV-194_	ESBL	aminopenicillins, cephalosporins	1	3.1
*bla*_SHV-199_	ESBL	aminopenicillins, cephalosporins	1	3.1
*bla*_SHV-26_	b-lactamase	aminopenicillins	1	3.1
*bla*_SHV-78_	ESBL	aminopenicillins, cephalosporins	1	3.1
*cmlA1*	chloramphenicol efflux	phenicol	1	3.1
*bla*_SHV-41_	b-lactamase	aminopenicillins	1	3.1
*bla*_OXA-181_	carbapenemase	cephalosporins, carbapenems	1	3.1
*bla*_CMY-4_	b-lactamase	aminopenicillins	1	3.1

### β-Lactam resistance

**Figure 3:**
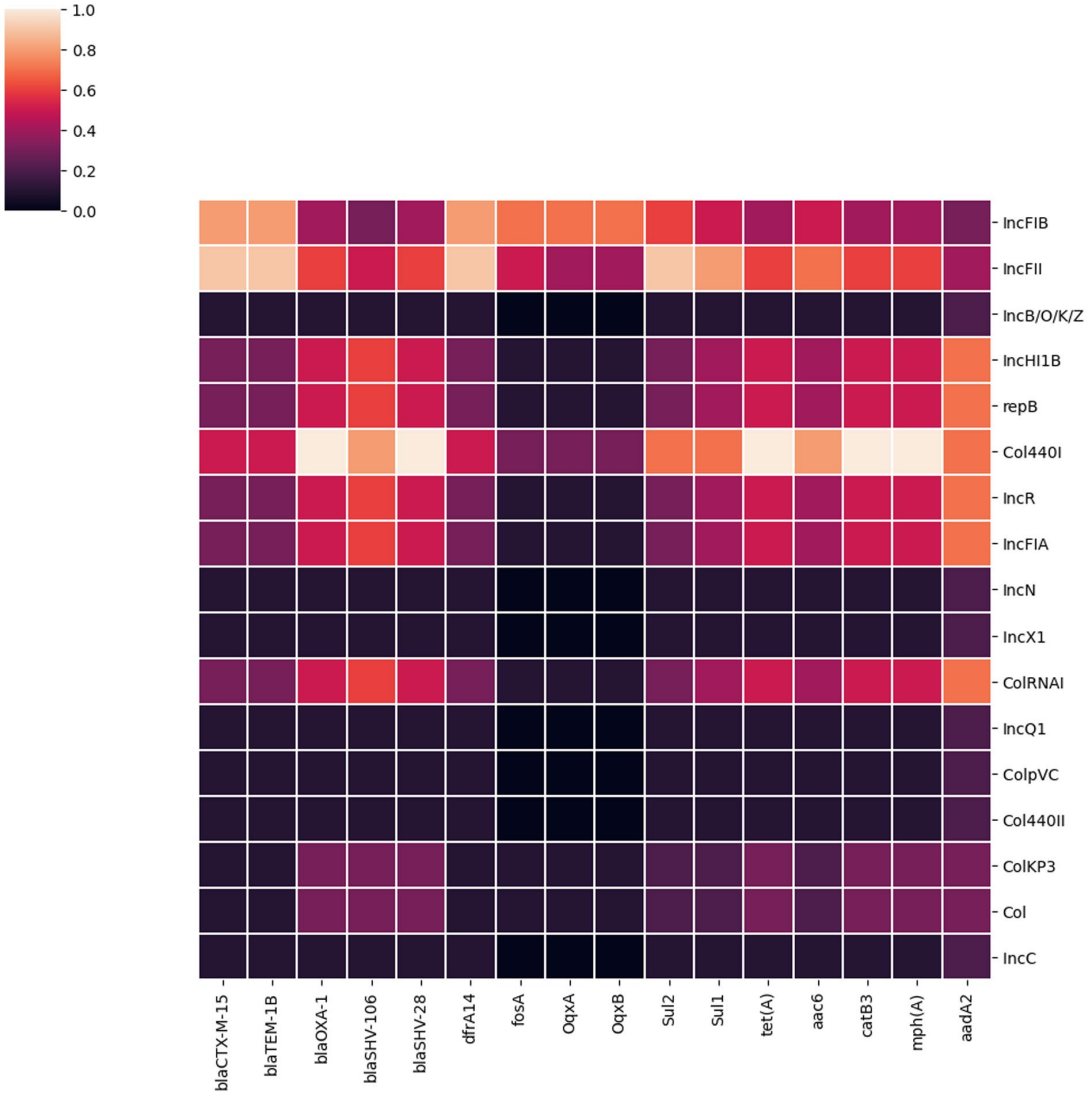
A heatmap illustrating the association between plasmid replicon types and the major AMR genes present in 32 *K. pneumoniae* isolates. It portrays that most AMR genes in *K. pneumoniae* isolates from Dhaka were associated with IncFIB, IncFII and Col440I plasmids.

We identified 35 genes conferring resistance to β-lactam antibiotics. Among all the identified β-lactam genes, *bla*_CTX-M-15_ (16/32) and *bla*_TEM-1B_ (16/32) were predominant. We also detected the presence of several allelic variants (29) of *bla*_SHV_ gene (Table 2). We observed a 100% concordance between the presence of ESBL genes (*bla*_CTX-M-15_ and *bla*_TEM-1B_) and cephalosporin resistance, particularly a significant association with ceftriaxone and ceftazidime antibiotics (*p* < 0.001). Sequence analysis identified that the *bla*_CTX-M-15_ genes were mostly present on plasmid DNA [14/16 (88%)] rather than on chromosomes [2/16 (13%)] (Table 3). It was majorly associated with three plasmid replicons, IncFII, followed by IncFIB and Col440I (Figure 3). The genetic environment of *bla*_CTX-M-15_ in most strains comprises the insertion element IS*Ecp1*. Other insertion sequences identified were IS*Kra4* and IS*6100* adjacent to the *bla*_CTX-M-15_ gene. All *bla*_CTX-M-15_ positive strains demonstrated the MDR phenotype and were affiliated with diverse sequence types (STs). We detected three genes encoding carbapenem resistance, including *bla*_NDM-5_ (6%)_,_ *bla*_OXA-232_ (6%) and *bla*_OXA-181_ (3%)_._ Two strains concomitantly harbored both *bla*_NDM-5_ and *bla*_OXA-232_ genes. One strain harbored the *bla*_OXA-181_ gene. All three strains carrying carbapenem resistance genes showed a difficult-to-treat resistance (DTR) phenotype, as these strains were resistant to all β-lactams, together with carbapenems and fluoroquinolone agents tested. In fact, they were resistant to all antibiotics tested (19/20) except for colistin. These three strains also coharbored *bla*_CTX-M-15_ and *bla*_TEM-1B_ genes.

**Table 3:** Characteristics of CTX-M-15 associated clinical *K. pneumoniae* strains from Dhaka, Bangladesh.

Genome ID	Source	Isolation date	STs	Genome locus	Insertion sequence flanking *bla*_CTX-M-15_	Plasmid replicons
GCKp1	Urine	17-Aug-19	ST985	Plasmid	ISEcp1	IncFIB(K), IncFII(K)
GCKp2	Urine	17-Aug-19	ST985	Plasmid	ISEcp1	IncFIB(K), IncFII(K)
GCKp3	Urine	19-Aug-19	ST1307	Chromosome	ISEcp1	IncB/O/K/Z
GCKp9	Urine	25-Aug-19	ST231	Plasmid	None	Col440I, IncFIA, IncFIB, IncN, IncR, IncX1
GCKp12	Urine	30-Aug-19	ST420	Plasmid	ISEcp1	IncFII, IncHI1B, IncR, repB
GCKp13	Urine	28-Aug-19	ST35	Plasmid	ISKra4	Col440I, FIA
GCKp14	Urine	1-Sep-19	ST15	Chromosome	None	Col440I, ColRNAI, IncFIB, IncFIB, IncQ1
GCKp15	Urine	16-Sep-19	ST14	Plasmid	None	IncFIB(K), IncFII(K)
GCKp18	Urine	18-Sep-19	ST101	Plasmid	None	Col440I, Col440II FIA, IncFIB, IncFII
GCKp20	Urine	25-Sep-19	ST420	Plasmid	None	IncFII, IncHI1B, repB
GCKp22	Urine	25-Sep-19	ST395	Plasmid	None	ColKP3, ColRNAI, IncFIB, IncFII
GCKp24	Urine	30-Sep-19	ST147	Plasmid	None	ColKP3, IncFII, IncFII(K), IncR
GCKp27	Urine	4-Oct-19	ST1427	Plasmid	ISEcp1	IncFIB(K)
GCKp28	Urine	2-Oct-19	ST211	Plasmid	IS6100	IncFII(K), IncR
GCKp30	Urine	7-Oct-19	ST307	Plasmid	ISEcp1	Col, IncFIB(K), IncFII(K)
GCKp31	Urine	13-Oct-19	ST11	Plasmid	None	ColRNAI, IncC, IncFIB, IncR

### Aminoglycoside resistance

We identified eight genes associated with aminoglycoside resistance. These include *aph(3″)-Ib* (37.5%)*, aph(6)-Id* (35%)*, aac(6′)-Ib-cr* (31.3%)*, aadA2* (18.8%)*, aac(3)-IIa* (15.6%)*, rmtF* (9.4%)*, aph(3′)-Ia* (6.3%) *and armA* (6.3%). Out of these eight genes, the gene *aac(6′)-Ib-cr* was significantly associated with the gentamicin resistance phenotype (*p* < 0.001). There was no association between these genes and amikacin resistance phenotype.

### Fluoroquinolone resistance

We screened for mutations in genes responsible for quinolone resistance. We detected substitutions at codon positions S80I (six isolates), S83Y (two isolates) in the amino acid sequence of *parC* and S83I (two isolates), S83F (three isolates) and D87A (five isolates) in the amino acid sequence of *gyrA*. Seven isolates had mutations in both the quinolone resistance genes *gyrA* and *ParC*. The ciprofloxacin and nalidixic acid resistance was associated with all the above-identified mutations in *gyrA* and *parC*. In addition to this we identified acquired AMR genes encoding for fluoroquinolone resistance such as *oqxA*/*oqxB* [32/32 (100%) isolates], *qnrB1* [6/32 (3.5%) isolates] and *qnrS* [6/32 (3.5%) isolates].

### Plasmid incompatibility groups

We identified 17 different plasmid incompatibility groups among 32 *K. pneumoniae* isolates (including FIB, FII, B/O/K/Z, Col44I, HI1B, repB, R, FIA, N, X1, ColRNA, Q1, ColPVC, Col440II, ColKP3, ColC) (Figure 3). FIB (66%), FII (44%) and Col440I (25%) were the predominant plasmid replicons across all 32 isolates. Consequently, the majority of AMR genes were associated with these three plasmid replicons (FIB, FII and Col440I) as shown in Figure 3.

### Virulome of *Klebsiella pneumoniae* strains

**Figure 4:**
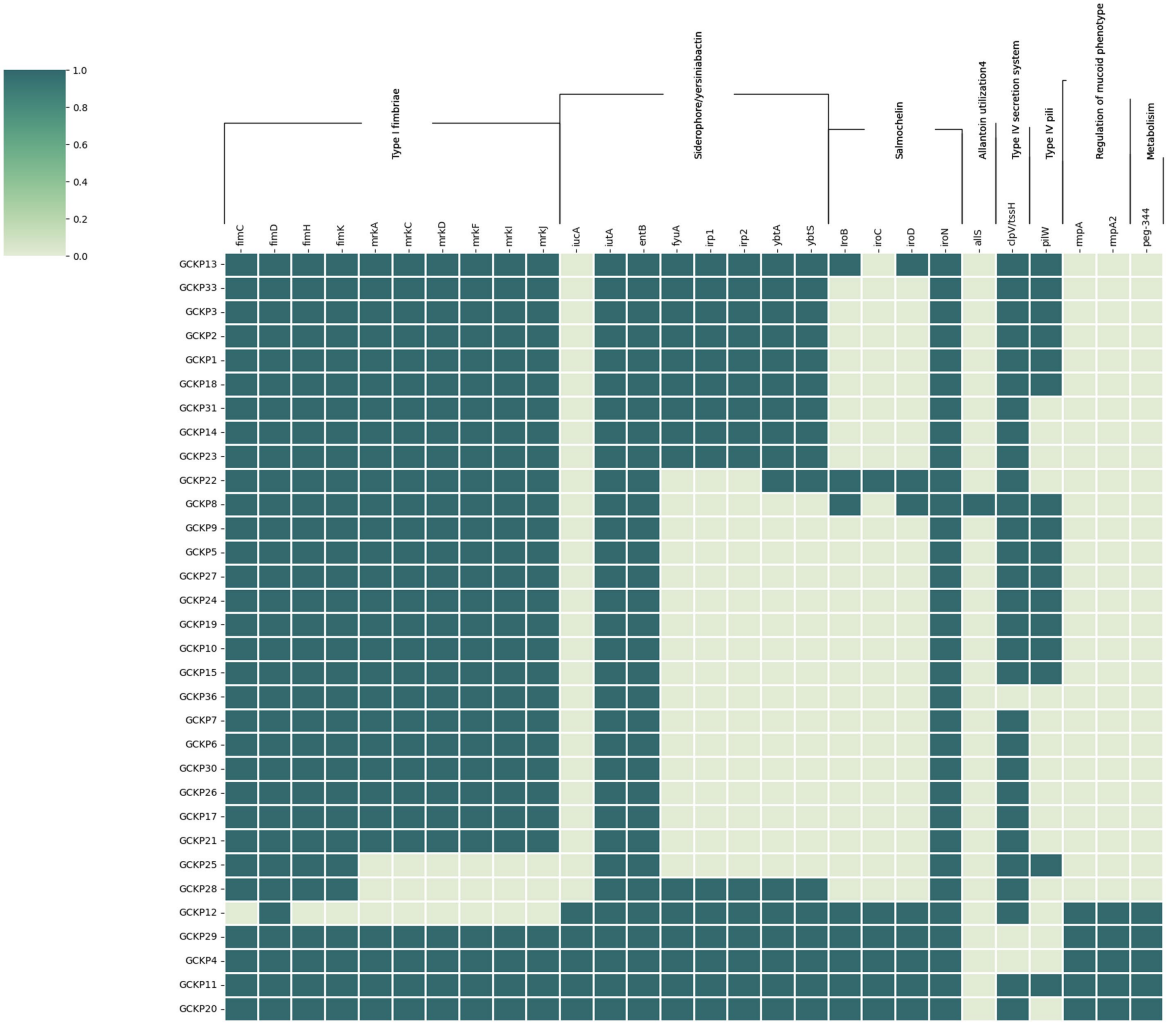
The virulome of 32 *K. pneumoniae* isolates from Dhaka: the virulence genes (*n* = 28) in *K. pneumoniae* were screened using VFDB and BLASTn analysis of known targets. The dark colored blocks indicate the presence of virulence genes.

Figure 4 shows the distribution of virulence factors associated with adherence, iron uptake, nutritional factor, secretion system, metabolism regulator, and mucoid phenotype among the 32 *K. pneumoniae* isolates. All (28) the virulence genes were detected in at least one *K. pneumoniae* isolate. Seven genes (*fimD*, *fimC*, *fimH*, *fimK*, *iutA*, *entB*, and *iroN*) exhibited prevalence rates of >90%. Six genes belonging to the type 3 fimbriae (*mrkA*, *mrkC*, *mrkD*, *mrkF*, *mrkI*, and *mrkJ*) and a gene belonging to type IV secretion system (*clp/tssH*) exhibited >80% prevalence and the rest of the genes exhibited <50% prevalence rates. For the four salmochelin genes, the order was: *iroN* (97%), *iroB* (22%), *iroD* (22%) and *iroC* (16%). Further, according to the aforementioned criteria, 6 (19%) strains in our collection were qualified as hypervirulent *K. pneumoniae* (hvKp) strains. Consequently, the remaining 26 strains were denoted as classical *K. pneumoniae* strains (cKp). Five out of the six hvKp strains were associated with all the five biomarkers of hypervirulence [*peg-344* (metabolite transporter), *iroB*; *iucA* (siderophores) *p*-*rmpA*; *p*-*rmpA2* (regulator of mucoid phenotype)]. One hvKp strain (GCKP8) harbored only one (*iroB*) hvKp-associated biomarker. One hvkp strain (GCKP11) was also positive for colibactin gene (*pks* gene cluster) rest of the strains (31 strains) were all negative for colibactin gene. Interesting, among the six hvKp strains, we identified two MDR (*bla*_CTX-M-15_ -positive) hypervirulent *K. pneumoniae* (MDR-hvKp) strains, while four (4/6) of the hvKp strains had non-MDR phenotypes. The two MDR-hvKp strains belonged to ST 420, serotype O1 antigen, and K20 capsular type. In contrast to the cKP strains, the hvKp strains had higher virulence prevalence, as the aggregate virulence score [median (range)] for hvKp strains [25 (18–27)] was higher than the cKp strains [15 (9–20)]. Moreover, the mean rank of virulence genes was significantly higher in hvKp strains than in cKp strains (*p* < 0.05).

## Discussion

*Klebsiella pneumoniae* is a significant pathogen worldwide because of its link with a growing number of infections and high rates of AMR. It is a predominant community and hospital-acquired pathogen often associated with high-risk patients. However, data on the genomics epidemiology of such strains from Bangladesh is very sparse. To expand our understanding of the genomics of *K. pneumoniae* in Dhaka, Bangladesh, we performed an exhaustive molecular characterization of 32 *K. pneumoniae* clinical isolates obtained from a referral diagnostic center at icddr,b, Dhaka, Bangladesh, between August to October 2019. We used a genomics-based approach to analyze the genetic diversity/relatedness, antimicrobial resistance and the virulome of the study isolates.

We have shown that the population of *K. pneumoniae* isolates from Bangladesh reflects the global population structure of *K. pneumoniae*. However, the isolates from this study exhibited only 2 of 3 *K. pneumoniae* lineages comprising KpI and KpII. The KpIII lineage was not detected in our collection; this could be because the study isolates were sampled from a single site and for a short time frame (3 months). However, an earlier study has reported the outbreak of multi-drug resistant infections of KpIII lineage (*K. variicola*) in Bangladesh (Farzana et al., 2019). The pan-genome and core genome analysis revealed reduced nucleotide diversity in the study isolates.

In this study, 26 STs were identified in the genomes analyzed. In particular, we have identified six international high-risk multidrug-resistant clones consisting of ST14, ST15, ST11, ST307, ST231, and ST147. Globally, several studies have reported that these STs were associated with high rates of AMR, including MDR and extensively drug-resistant (XDR) strains of *K. pneumoniae* (Navon-Venezia et al., 2017; Mancini et al., 2018). The predominance of well-known international high-risk clones, including the clonal groups CG258 (ST11) and CG15 (ST14, ST15), is not unique to this study. However, in a previous study, some of these clones were described to be associated with hospital-acquired infections among carbapenem-resistant strains (Okanda et al., 2021). Since no *a priori* selection of resistant phenotypes was performed in this study, this indicates a strong prevalence of high-risk clones among *K. pneumoniae* associated with community-acquired infections. All strains [25% (8/32)] affiliated with these high-risk clones were found to be MDR and harbored ESBL genes such as *bla*_CTX-M-15_ and/or *bla*_TEM-1B,_ except for one strain of ST14. The plasmid replicons FIA and FII were enriched in these strains.

In addition to the high-risk multidrug-resistant clones described above, we identified six (19%) hypervirulent *K. pneumoniae* (hvKp) strains in our collection. hvKp strains have gained increased attention globally, as these often infect healthy individuals from the community causing invasive and metastatic infections (Decré et al., 2011; Pomakova et al., 2011; Siu et al., 2011). The hvKp strains in this study exhibited high virulence scores compared to the classical *K. pneumoniae* (cKp) strains. The six hvKp strains represented all 3 months of the sampling period. These six hvKp strains comprised of 2 ST 420: K20, 2 ST65: K2, 1 ST268: K20 and 1 ST4321: K39. Previous studies have reported the presence of hvKp strains among 3 of 4 STs (ST420, ST65 and ST268) identified in this study (Shen et al., 2020; Eger et al., 2021). However, the strain with ST4321 was assigned as hvKp in this study, harboring only one hypervirulence biomarker. The presence of hypervirulence biomarker in sequence type ST4321 described here indicates that hvKp strains are evolving across various clonal types. The majority of the hvKp strains were affiliated with O1 antigen and K20 capsular polysaccharides. IncHI1B and repB plasmid replicons were predominant in hvKp strains. The two hvKp strains associated with ST420 were MDR and positive for ESBL genes (*bla*_CTX-M-15_ and *bla*_TEM-1B_). This is, to the best of our knowledge, the first report of hvKp strains in Bangladesh.

Another significant group of strains observed in this study comprised three *K. pneumoniae* strains (9%) that were positive for carbapenem resistance genes. Two of the three carbapenem-resistant strains were concomitantly positive for *bla*_NDM-5_ and *bla*_OXA-232_ and another strain was positive for *bla*_OXA-181_ (a variant of *bla*_OXA48_). All these three strains were extensively drug-resistant (XDR), exhibiting a difficult-to-treat phenotype. The three strains belonged to ST395:K64, ST147:K10 and ST11:K24. Earlier studies described these three STs harboring carbapenem resistance genes as epidemic clones responsible for significant morbidity and mortality (Logan and Weinstein, 2017). The three strains exhibited an average of 18 (out of 28 genes tested) virulence genes representing type I fimbriae, siderophores/yersiniabactin, salmochelin and type IV secretion system, indicating grave consequences of infection with such strains.

In general, the ESBL genotypes were also prevalent in strains other than the high-risk clones and carbapenem-resistant strains, indicating high propensities of *K. pneumoniae* to acquire and spread ESBL genes. We found a link between the presence of key AMR genes and a small number of plasmid replicons, such as IncFIB, IncFII and Col440I. This suggests that a few plasmids can play an important role in spreading AMR genes. Especially, the genetic mechanisms for acquiring and spreading ESBL and carbapenem resistance are widespread in the study setting (Mazumder et al., 2020a, 2021). Any selective pressure exerted by the indiscriminate use of a reserve group of antibiotics would lead to the prevalence of XDR strains in the population. Overall, the phylogenetic analysis and STs identified revealed a heterogenous distribution of the *K. pneumoniae* strains. The lone strain belonging to the KpII lineage (GCKP36) (*K. quasipneumoniae*) was susceptible to most antibiotics and consequently had fewer resistance genes. The strain was untypeable for MLST and K types and had a moderate number of virulence genes. This strain was misidentified as *K. pneumoniae* using the Vitek-2 system. However, a recent report has shown that strains of *K. quasipneumoniae* were no less virulent than *K. pneumoniae* strains (Long et al., 2017).

The first limitation of this investigation is that the Dhaka isolates originated from a single location. The second limitation is that a small number of isolates were sampled for a short duration (3 months only). The third limitation is that the hypervirulence is determined only on the basis of molecular markers previously reported. These limitations prevent the generalizability of the study findings to the country level. However, the parallels between our findings and those of other research in the region (Sherchan et al., 2020; Sundaresan et al., 2022) including the similarities in population structure, emergence of resistant and hypervirulent lineages indicate that this study is most probably representative of the genomic epidemiology of *K. pneumoniae* in Bangladesh. The main advantage of this study is that we have not selected the isolates intending to enrich for resistance or virulence phenotypes or genotypes. This approach allowed us to describe the population structure and various features associated with *K. pneumonia* in the community-acquired infections in this setting.

In conclusion, our analysis of 32 genomes of *K. pneumoniae* revealed the abundance of ESBL *K. pneumoniae*. We have shown that the *K. pneumoniae* population in Dhaka, Bangladesh is similar to other settings globally. We strongly suggest the detection of several major high-risk multidrug-resistant clonal lineages such as ST14, ST15, ST11, ST307, ST231 and ST147 in our collection. We confirm a moderately high prevalence (19%) of hypervirulent *K. pneumoniae* (hvKp), which is of particular concern that needs to be addressed immediately. The presence of difficult-to-treat carbapenem-producing *K. pneumoniae* strains needs more attention to prevent its dissemination. The consistency in population structure (prevalence of high-risk and hypervirulent lineages) with global isolates puts Bangladesh in the global context in terms of exchange of significant epidemiological *K. pneumoniae* clones, and these may cause untreatable aggressive infections in a country where the health care systems have very limited antibiotic options available for treatment.

The hypervirulent *K. pneumoniae* (hvKp) report and the over-representation of the international high-risk multidrug-resistant clones warrant WGS-based surveillance to closely monitor the evolutionary trends and convergence of virulence and resistance among *K. pneumoniae* strains in Bangladesh.

## Data availability statement

The datasets presented in this study can be found in online repositories. The names of the repository/repositories and accession number(s) can be found at: https://www.ncbi.nlm.nih.gov/, JACSGO000000000.1, https://www.ncbi.nlm.nih.gov/, JACSGN000000000.1, https://www.ncbi.nlm.nih.gov/, JACSGM000000000.1, https://www.ncbi.nlm.nih.gov/, JACSGL000000000.1, https://www.ncbi.nlm.nih.gov/, JACSGK000000000.1, https://www.ncbi.nlm.nih.gov/, JACSGJ000000000.1, https://www.ncbi.nlm.nih.gov/, JACSGI000000000.1, https://www.ncbi.nlm.nih.gov/, JACSGH000000000.1, https://www.ncbi.nlm.nih.gov/, JACSGG000000000.1, https://www.ncbi.nlm.nih.gov/, JACSGF000000000.1, https://www.ncbi.nlm.nih.gov/, JACSGE000000000.1, https://www.ncbi.nlm.nih.gov/, JACSGD000000000.1, https://www.ncbi.nlm.nih.gov/, JACSGC000000000.1, https://www.ncbi.nlm.nih.gov/, JACSGB000000000.1, https://www.ncbi.nlm.nih.gov/, JACSGA000000000.1, https://www.ncbi.nlm.nih.gov/, JACSFZ000000000.1, https://www.ncbi.nlm.nih.gov/, JACSFY000000000.1, https://www.ncbi.nlm.nih.gov/, JACSFX000000000.1, https://www.ncbi.nlm.nih.gov/, JACSFW000000000.1, https://www.ncbi.nlm.nih.gov/, JACSFV000000000.1, https://www.ncbi.nlm.nih.gov/, JACSFU000000000.1, https://www.ncbi.nlm.nih.gov/, JACSFT000000000.1, https://www.ncbi.nlm.nih.gov/, JACSFS000000000.1, https://www.ncbi.nlm.nih.gov/, JACSFR000000000.1, https://www.ncbi.nlm.nih.gov/, JACSFQ000000000.1, https://www.ncbi.nlm.nih.gov/, JACSFP000000000.1, https://www.ncbi.nlm.nih.gov/, JACSFO000000000.1, https://www.ncbi.nlm.nih.gov/, JACSFN000000000.1, https://www.ncbi.nlm.nih.gov/, JACSFM000000000.1, https://www.ncbi.nlm.nih.gov/, JACSFL000000000.1, https://www.ncbi.nlm.nih.gov/, JACSFK000000000.1, https://www.ncbi.nlm.nih.gov/, JACSFI000000000.1.

## Author contributions

AH and RM designed the study, drafted the manuscript, and performed genome sequencing. AH, AA, and RM carried out the bioinformatics analyzes and interpretation of results and prepared tables and figures. US provided technical assistance in microbiology and sequencing work. JP, SC, MA, DA, TC, and DM contributed to the discussions and reviewed the manuscript. DM supervised the study. All authors have read and approved the final manuscript.

## Conflict of interest

The authors declare that the research was conducted in the absence of any commercial or financial relationships that could be construed as a potential conflict of interest.

## Publisher’s note

All claims expressed in this article are solely those of the authors and do not necessarily represent those of their affiliated organizations, or those of the publisher, the editors and the reviewers. Any product that may be evaluated in this article, or claim that may be made by its manufacturer, is not guaranteed or endorsed by the publisher.
